# Association Between Schizophrenia-Related Polygenic Liability and the Occurrence and Level of Mood-Incongruent Psychotic Symptoms in Bipolar Disorder

**DOI:** 10.1001/jamapsychiatry.2017.3485

**Published:** 2017-11-22

**Authors:** Judith Allardyce, Ganna Leonenko, Marian Hamshere, Antonio F. Pardiñas, Liz Forty, Sarah Knott, Katherine Gordon-Smith, David J. Porteous, Caroline Haywood, Arianna Di Florio, Lisa Jones, Andrew M. McIntosh, Michael J. Owen, Peter Holmans, James T. R. Walters, Nicholas Craddock, Ian Jones, Michael C. O’Donovan, Valentina Escott-Price

**Affiliations:** 1 Medical Research Council Centre for Neuropsychiatric Genetics and Genomics, Institute of Psychological Medicine and Clinical Neurosciences, School of Medicine, Cardiff University, Cardiff, Wales; 2 Department of Psychological Medicine, University of Worcester, Worcester, England; 3 Medical Genetics Section, Centre for Genomic and Experimental Medicine, Institute of Genetics and Molecular Medicine, University of Edinburgh, Edinburgh, Scotland; 4 Division of Psychiatry, University of Edinburgh, Edinburgh, Scotland

## Abstract

**Importance:**

Bipolar disorder (BD) overlaps schizophrenia in its clinical presentation and genetic liability. Alternative approaches to patient stratification beyond current diagnostic categories are needed to understand the underlying disease processes and mechanisms.

**Objective:**

To investigate the association between common-variant liability for schizophrenia, indexed by polygenic risk scores (PRSs), and psychotic presentations of BD.

**Design, Setting, and Participants:**

This case-control study in the United Kingdom used multinomial logistic regression to estimate differential PRS associations across categories of cases and controls. Participants included in the final analyses were 4436 cases of BD from the Bipolar Disorder Research Network. These cases were compared with the genotypic data for 4976 cases of schizophrenia and 9012 controls from the Type 1 Diabetes Genetics Consortium study and the Generation Scotland study. Data were collected between January 1, 2000, and December 31, 2013. Data analysis was conducted from March 1, 2016, to February 28, 2017.

**Exposures:**

Standardized PRSs, calculated using alleles with an association threshold of *P* < .05 in the second Psychiatric Genomics Consortium genome-wide association study of schizophrenia, were adjusted for the first 10 population principal components and genotyping platforms.

**Main Outcomes and Measures:**

Multinomial logit models estimated PRS associations with BD stratified by Research Diagnostic Criteria subtypes of BD, by lifetime occurrence of psychosis, and by lifetime mood-incongruent psychotic features. Ordinal logistic regression examined PRS associations across levels of mood incongruence. Ratings were derived from the Schedules for Clinical Assessment in Neuropsychiatry interview and the Bipolar Affective Disorder Dimension Scale.

**Results:**

Of the 4436 cases of BD, 2966 (67%) were female patients, and the mean (SD) age at interview was 46 [12] years. Across clinical phenotypes, there was an exposure-response gradient, with the strongest PRS association for schizophrenia (risk ratio [RR] = 1.94; 95% CI, 1.86-2.01), followed by schizoaffective BD (RR = 1.37; 95% CI, 1.22-1.54), bipolar I disorder subtype (RR = 1.30; 95% CI, 1.24-1.36), and bipolar II disorder subtype (RR = 1.04; 95% CI, 0.97-1.11). Within BD cases, there was an effect gradient, indexed by the nature of psychosis. Prominent mood-incongruent psychotic features had the strongest association (RR = 1.46; 95% CI, 1.36-1.57), followed by mood-congruent psychosis (RR = 1.24; 95% CI, 1.17-1.33) and BD with no history of psychosis (RR = 1.09; 95% CI, 1.04-1.15).

**Conclusions and Relevance:**

For the first time to date, a study shows a polygenic-risk gradient across schizophrenia and BD, indexed by the occurrence and level of mood-incongruent psychotic symptoms.

## Key Points

**Question:**

What is the association between schizophrenia-related polygenic liability and the occurrence and level of mood-incongruence of psychotic symptoms in bipolar disorder?

**Findings:**

In this case-control study involving 4436 cases of bipolar disorder, 4976 cases of schizophrenia, and 9012 controls, there was an exposure-response gradient of polygenic risk. Schizophrenia had the strongest association, followed by bipolar disorder with prominent mood-incongruent psychotic features, bipolar disorder with mood-congruent psychotic features, and bipolar disorder with no psychosis; all differential associations were statistically significant.

**Meaning:**

This study shows a gradient of genetic liability across schizophrenia and bipolar disorder, indexed by the occurrence of psychosis and level of mood incongruence.

## Teaser

This case-control study examines the association between polygenic liability for schizophrenia and psychotic presentations of bipolar disorder.

## Introduction

Although classified as a discrete diagnostic category,^1^ bipolar disorder (BD) overlaps considerably with schizophrenia (SCZ) in both its clinical presentation^4^ and genetic liability.^14^ Bipolar disorder is a phenomenologically heterogeneous construct, and within the diagnostic category, individuals with BD may have quite different symptom profiles. It has been proposed that this clinical heterogeneity indicates underlying etiological heterogeneity and that the degree of clinical similarity between BD and SCZ reflects overlapping alleles, which selectively influence specific, shared clinical characteristics rather than the global risk for the disorder.^23^

Delusions and hallucinations are common in BD,^26^ with approximately one-third of all psychotic features judged to be mood incongruent.^28^ Mood-incongruent psychotic features are associated with poor prognosis and poor lithium response and are qualitatively similar to the prototypic symptoms of SCZ,^30^ suggesting that BD with psychosis and particularly mood-incongruent psychotic features may specify a subgroup or stratum with stronger etiological links to SCZ. Stratified linkage and candidate-gene studies of BD associations with chromosomal regions and genes implicated in SCZ show stronger effects in psychosis and mood-incongruent subsamples,^33^ providing some support for this causal heterogeneity hypothesis; however, lack of consistency in earlier linkage and candidate-gene studies renders the overall support weak.

Genome-wide association studies (GWAS) have found a substantial polygenic component to both BD and SCZ risks, with a large proportion of the disorders’ genetic variance explained by common alleles partially shared by the 2 disorders.^20^ This polygenic risk can be calculated for individuals with a single summary measure: the polygenic risk score (PRS; with higher scores indicating a higher burden of risk alleles), which allows us to examine the genetic basis of symptom domains within and across the 2 disorders^37^ with greater power than do the historical linkage and candidate-gene approaches. The PRS-SCZ differentiates BD cases from controls,^16^ and there are differential PRS associations across subtypes with schizoaffective bipolar disorder (SABD) (an intermediate subtype characterized by admixture of SCZ and BD symptoms) having a relatively larger burden of SCZ risk, compared with other BD subtypes.^15^ To date, lack of power in well-phenotyped samples has hindered fine-scale examination of the association of SCZ polygenic-risk with psychotic symptoms in BD.

This study aimed to examine the association between polygenic liability for SCZ and psychotic presentations of BD using the PRSs generated from the most powerful SCZ-GWAS discovery set currently available.^21^ Measures relevant to the occurrence and nature of psychotic symptoms were considered. We hypothesized that BD with psychosis would be associated with higher polygenic risk for SCZ and that this association would be stronger when mood-incongruent psychotic features were present, given their phenotypic similarity to the psychotic symptoms of prototypic SCZ.

## Methods

### Sample Ascertainment

#### Bipolar Disorder Sample

In total, data from 4436 cases of BD with deep phenotypic information, European ancestry, and domicile in the United Kingdom were collected between January 1, 2000, and December 31, 2013, via the UK Bipolar Disorder Research Network using recruitment methods reported previously.^15^ The sample contained 1399 cases not included in previous publications of the Bipolar Disorder Research Network.^15^ All participants were assessed using a consistent protocol, which included the Schedules for Clinical Assessment in Neuropsychiatry (SCAN) interview^43^ administered by trained research psychologists and psychiatrists, with very good to excellent interrater reliability for all domains of psychopathology.^44^ Using information from the SCAN interview and case note review, we completed the Operational Criteria Checklist.^45^ Research Diagnostic Criteria (RDC)^3^ diagnoses, which differentiate individuals on the basis of their pattern of mood and psychotic symptoms better^40^ than either the *DSM-5*^2^ or the *International Statistical Classification of Diseases and Health-Related Disorders*, *Tenth Revision* Classification of Mental and Behavioural Disorders,^1^ were made with consensus lifetime best-estimate method informed by all available information.^46^ The Bipolar Disorder Research Network study was given a favorable ethical opinion by the West Midlands Multi-Centre Research Ethics Committee. Local research and development approval was obtained in all participating National Health Service Trusts and Health Boards. All participants gave written informed consent. Data analysis was conducted from March 1, 2016, to February 28, 2017.

#### Schizophrenia Sample

To allow the comparison of BD to SCZ, we included a subset (n = 4976) of the CLOZUK (treatment-resistant schizophrenia, treated with clozapine) study sample collected via the Zaponex Treatment Access System, which was detailed in a previous report.^47^ All patients in the sample were prescribed clozapine for treatment-resistant SCZ and are independent of and unrelated (pi-hat <0.2) with individuals in the discovery GWAS.^21^ In principle, treatment-resistant SCZ may carry higher polygenic risk burden; however, the PRSs in the CLOZUK sample are similar to the PRSs in other SCZ samples used by the Psychiatric Genomics Consortium.^21^ The CLOZUK procedures and methods were approved by the National Research Ethics and were in line with the UK Human Tissue Act regulations in partnership with the Leyden Delta.

#### Control Samples

The controls came from 2 UK sources: (1) the Type 1 Diabetes Genetics Consortium study, which comprised unscreened controls (n = 2532) recruited through the 1958 Birth Cohort,^48^ and (2) a subsample (n = 6480) of the Generation Scotland study screened for psychiatric disorders.^49^ Controls were not associated (pi-hat <0.2) with individuals in the Psychiatric Genomics Consortium-SCZ discovery set and were matched ancestrally to our case data sets.^47^ The Generation Scotland Access Committee approved this application to use Generation Scotland as controls.

### Genotyping, Quality Control, Phasing, and Imputation

#### Bipolar Cases

Genotypic data for the BD cases were processed in 3 batches, each on a different platform. To mitigate against potential bias from batch effects,^50^ stringent quality control (QC) was performed on each platform separately prior to merging. Single-nucleotide polymorphisms (SNPs) were excluded if the call rate was less than 98%, the minor allele frequency (MAF) was less than 0.01, or the SNPs deviated from the Hardy-Weinberg equilibrium (HWE) at *P* < 1 × 10^−6^. Individuals were excluded if they had minimal or excessive autosomal homozygosity (*F*|>0.1), high pairwise relatedness (pi-hat >0.2), or mismatch between recorded and genotypic sex. Following QC, the data for each platform were phased using SHAPEIT,^51^ version 3.4.0.1023 (Olivier Delaneau), and imputed with IMPUTE2,^52^ version 2.3.0 (University of Oxford), using the 1000 Genomes Project reference panel (phase 3). Imputed data were converted into the most probable genotypes (probability >0.9) and merged on shared SNPs. After QC, 4399 BD cases remained.

#### CLOZUK Cases and Controls

The CLOZUK and control samples went through strict QC separately before being phased and imputed simultaneously as part of a larger SCZ study.^47^

### Merging Imputed Genotypic Data Sets

After SNPs with stand ambiguity were excluded, BD, CLOZUK, and control samples were merged and the imputed markers underwent a second QC filter.^50^ This second QC excluded SNPs with a missingness rate of more than 5% of individuals, an information content score lower than 0.8, an MAF of less than 0.01, or deviation from HWE at *P* < 1 × 10^−6^.

### Principal Component Analysis

To adjust for potential confounding from population structure, we performed principal components analysis. We used PLINK, version 1.9 (Christopher Chang), after pruning the linkage disequilibrium and frequency filtering the SNPs from the merged sample, keeping the eigenvectors for the first 10 principal components to use as covariates in the association analysis.

### Polygenic Risk Scores

We generated the PRSs^20^ using the 2014 Psychiatric Genomics Consortium-SCZ meta-analysis as our discovery set^21^ calculated for each individual on the basis of a set of alleles with association *P* < .05. This decision was informed by the Psychiatric Genomics Consortium leave-one-cohort-out PRS analyses for all SNP selection *P* value thresholds, which found the median and the mode was *P* = .05, which represents the association that best optimizes the balance of false and true risk alleles at the current discovery sample size.^21^ The most informative and independent markers were selected to minimize statistical noise where possible, by using *P* value–informed clumping at *r^2^* < 0.2 with 1-MB windows and by excluding the extended major histocompatibility complex (chromosome 6: position 25-35 MB) because of its complex linkage disequilibrium structure.

### Outcome Measure of Lifetime Psychosis and Mood Incongruence

#### Subtypes of BD

The RDC subtypes were used as categorical outcomes in case-control analyses. The RDC^3^ and the *DSM-5*,^2^ although not the *International Statistical Classification of Diseases and Health-Related Disorders*, *Tenth Revision, Classification of Mental and Behavioural Disorders*,^1^ subdivides BD into bipolar I disorder (BD I) and bipolar II disorder (BD II) depending on the nature of the mood states, mania in BD I, and hypomania in BD II. All classification systems recognize SABD. Psychotic symptoms are most prominent in SABD and then BD I and are least prominent in BD II.^53^

#### Bipolar Affective Disorder Dimension Scale

Outcome measures were generated from the Bipolar Affective Disorder Dimension Scale (BADDS) subscales of psychosis and mood incongruence, which provide an ordered, but not necessarily linear, measure of lifetime symptom domain severity.^55^ An interrater reliability exercise for this sample demonstrates excellent interclass correlation: (psychosis) 0.91 and (mood incongruence) 0.89.

1. A binary categorical outcome measure for lifetime occurrence of psychosis, defined as an unambiguous episode of positive and/or disorganized psychotic symptoms, generated by dichotomizing the psychosis domain scale at a score higher than 9.^55^
2. A binary categorical outcome measure for lifetime occurrence of predominant mood-incongruent psychotic features, defined as high or low prominence of mood incongruence, generated by dichotomizing the mood incongruence domain scale at a score higher than 19.
3. An ordinal measure of mood-incongruent psychotic features that assesses the overall balance between mood-congruent and mood-incongruent psychosis across the lifetime, rated using all available information according to the Bipolar Disorder Research Network protocol (see eNote 1 in the eAppendix in the Supplement).

### Statistical Analysis

A multinomial logit model was used to estimate differential associations of standardized PRSs, adjusted for the first 10 principal components and genotyping platforms across the categories of cases and controls. We report the estimated coefficient transformed to relative risk ratio (RR), defined as the exponentiated regression coefficient. In addition, PRS associations across levels of mood-incongruent psychotic features using ordinal logistic regression were estimated. To examine whether SABD subtypes were driving observed PRS associations with mood-incongruent psychotic features, we did a sensitivity analysis that excluded SABD cases. Postestimation predicted probabilities were plotted to aid the interpretation of PRS associations across RDC subtypes of BD.^56^ To correct for multiple comparisons of PRS associations across different phenotypic strata within each model, we generated bootstrapped SEs and 95% CIs as an approximation to exact permutation methods^57^ (see eNote 2 in the eAppendix in the Supplement). Possible familywise, type I error proliferation was controlled using the Bonferroni method, calculated by multiplying the bootstrapped *P* values by 4.^58^

Post hoc analyses used a multinomial logit model case-control design to examine differential associations across composite phenotypic categories defined by BD I and BD II subtypes and stratified by psychosis status. Complementary logistic regression analyses were conducted to compare the PRS association with lifetime occurrence of psychosis across BD I and BD II subtypes. To examine the distribution of RDC-defined cases across PRS levels, we converted the PRSs to deciles and generated a stacked bar chart (SCZ [CLOZUK], SABD, BD I, BD II), by decile. Analyses were performed using PLINK, version 1.9 ^59^ (Christopher Chang), or Stata, version 14 (StataCorp, LLC).

## Results

### Sample Description, Genotyping, and Quality Control

Of the 4436 cases of BD, 2966 (67%) were female patients, and the mean (SD) age at the SCAN interview was 46 [12] years. After BD, CLOZUK, and control imputed-genotyped samples were merged and further QC was performed, 18 387 cases and controls (eTable 1 in the Supplement) with 3 451 354 SNPs, with an information content score higher than 0.8 and a MAF greater than 1%, were available for analysis. Within the BD sample, 2296 cases (52%) endorsed lifetime occurrence of definite psychosis, with less than a 1% missingness rate in this variable (n = 25). Of the BD cases with definite psychosis, 981 (43%) were classified as having high lifetime mood-incongruent psychotic features. There was a 9% missingness rate (n = 214) for the mood-incongruence variable within the BD cases with psychosis.

### Case-Control PRS Associations

As expected, the PRSs discriminated CLOZUK from control samples (Table 1). The PRSs in those with a diagnosis of SABD or BD I, but not BD II, were significantly higher than the PRSs in controls. Across clinical phenotypes, there was an exposure-response gradient, with the strongest PRS association for schizophrenia (RR = 1.94; 95% CI, 1.86-2.01), followed by schizoaffective BD (RR = 1.37; 95% CI, 1.22-1.54), BD I (RR = 1.30; 95% CI, 1.24-1.36), and BD II (RR = 1.04; 95% CI, 0.97-1.11).

**Table 1.** Differential Association of Polygenic Risk Scores Across Variously Defined Bipolar Disease Strata (Controls as Comparator Category)

Case	No. of Cases (Subsample)	Relative Risk Ratio^a^	Bootstrapped *P* Value	Bonferroni-Corrected *P* Value	Bootstrapped 95% CI
CLOZUK	4976	1.94	<.001	<.001	1.86-2.01
**Bipolar Disorder Cases Stratified by RDC-Defined Subtypes**					
SABD	356	1.37	<.001	<.001	1.22-1.54
BD I	2775	1.30	<.001	<.001	1.24-1.36
BD II	1268	1.04	.26	.26	0.97-1.11
**Bipolar Disorder Cases Stratified by LEP**					
No LEP	2079	1.09	.001	.004	1.04-1.15
LEP	2296	1.36	<.001	<.001	1.29-1.43
**Psychotic Bipolar Disorder Cases Stratified by Level of Mood Incongruence**					
Low LMI	1126	1.24	<.001	<.001	1.17-1.33
High LMI	981	1.46	<.001	<.001	1.36-1.57
**Sensitivity Analysis: Psychotic Bipolar Disorder Cases Stratified by Level of Mood Incongruence (Excluding SABD Cases)**					
Low LMI	1068	1.25	<.001	<.001	1.16-1.33
High LMI	699	1.49	<.001	<.001	1.37-1.62

Abbreviations: BD I, bipolar I disorder subtype; BD II, bipolar II disorder subtype; CLOZUK, treatment-resistant schizophrenia treated with clozapine study; LEP, lifetime ever occurrence of psychotic symptoms; LMI, lifetime pattern of low or high mood incongruent psychotic features; RDC, Research Diagnostic Criteria; SABD, schizoaffective bipolar disorder.

^a^ Adjusted for polygenic risk score for the first 10 principal components and genotyping platforms.

### PRS Associations Within Cases

**Figure 1.**
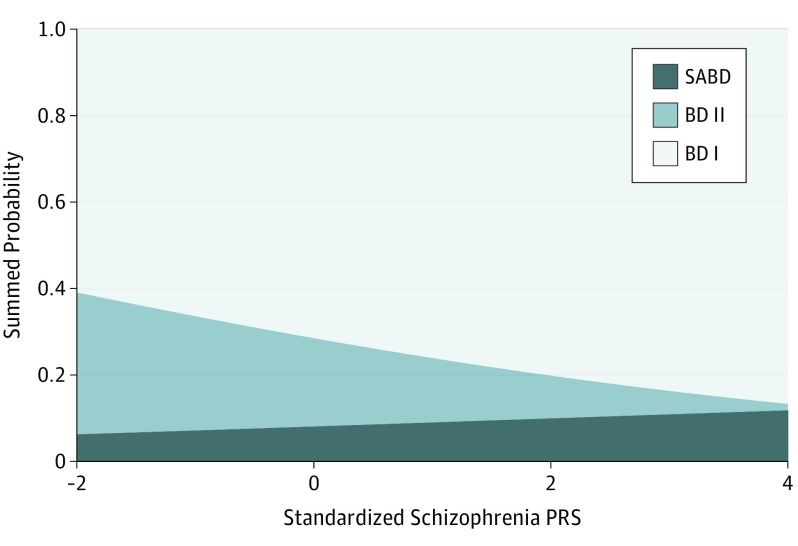
Probability of RDC Bipolar Subtype as a Function of Polygenic Risk Scores (PRSs) Associated With Schizophrenia BD I indicates bipolar I disorder subtype; BD II, bipolar II disorder subtype; and SABD, schizoaffective bipolar disorder.

**Figure 2.**
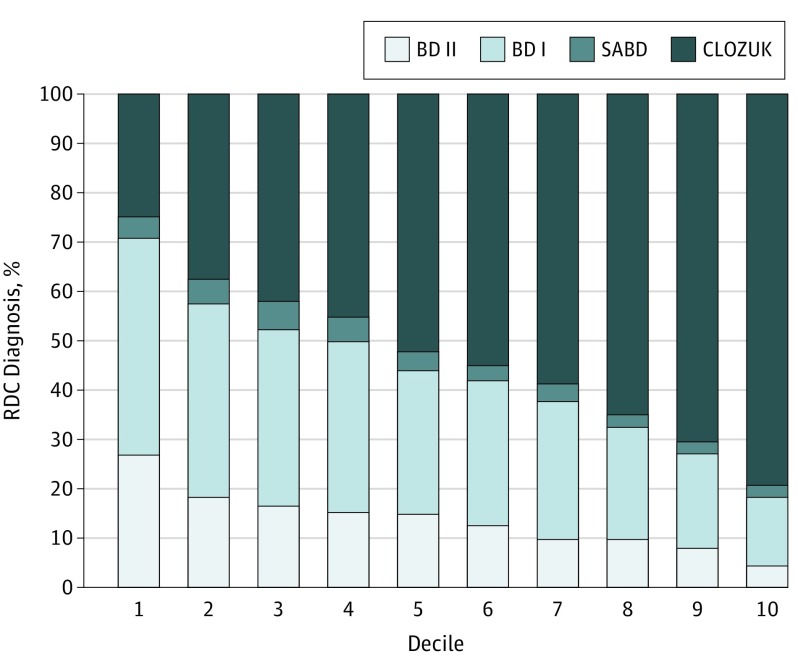
Percentage of Bipolar Subtype as a Function of Polygenic Risk Scores for Schizophrenia, Grouped by Decile BD I indicates bipolar I disorder subtype; BD II, bipolar II disorder subtype; CLOZUK, treatment-resistant schizophrenia treated with clozapine study; RDC, Research Diagnostic Criteria; and SABD, schizoaffective bipolar disorder.

The PRSs discriminated SCZ from all BD subtypes (Table 2). Within BD, the PRSs discriminated BD II from both BD I and SABD (Figure 1). The percentage of CLOZUK cases increased monotonically with increasing decile of PRS, while the percentage of bipolar subtypes decreased (Figure 2).

**Table 2.** Polygenic Risk Scores for Schizophrenia Associations Among Cases

Case	Relative Risk Ratio^a^	Bootstrapped *P* Value	Bonferroni-Corrected *P* Value	Bootstrapped 95% CI
SABD compared with TRS	0.71	<.001	<.001	0.63-0.80
BD I compared with TRS	0.67	<.001	<.001	0.64-0.71
BD II compared with TRS	0.54	<.001	<.001	0.50-0.57
SABD compared with BD II	1.32	<.001	<.001	1.16-1.50
BP I compared with BD II	1.25	<.001	<.001	1.16-1.35
SABD compared with BD I	1.05	.41	.41	0.93-1.18

Abbreviations: BD I, bipolar I disorder subtype; BD II, bipolar II disorder subtype; SABD, schizoaffective bipolar disorder; TRS, treatment-resistant schizophrenia.

^a^ Adjusted for polygenic risk score for the first 10 principal components and genotyping platforms.

### PRS Associations With Psychotic BD

**Figure 3.**
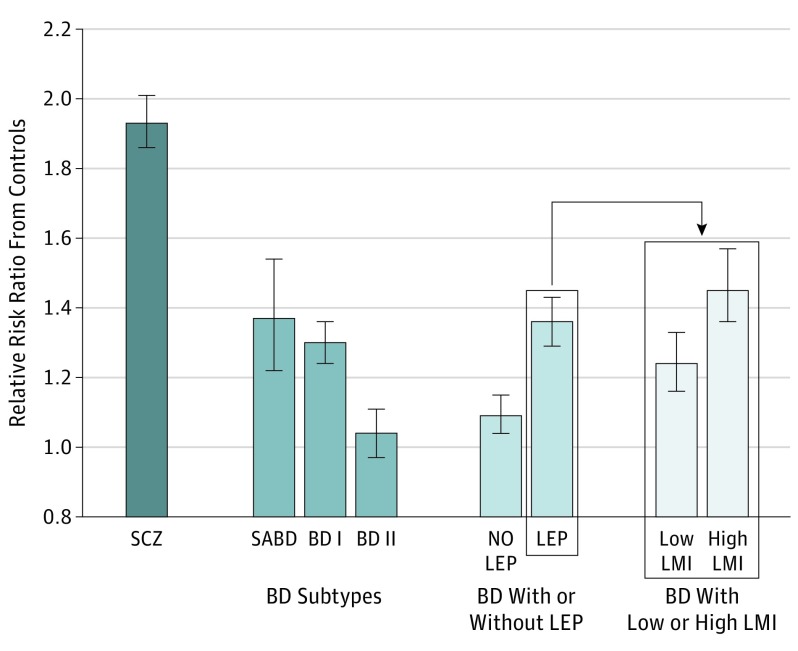
Relative Risk Ratios for Schizophrenia and Bipolar Subtypes The control group is the comparator. BD I indicates bipolar I disorder subtype; BD II, bipolar II disorder subtype; LEP, lifetime ever occurrence of psychotic symptoms; LMI, lifetime pattern of low or high mood incongruent psychotic features; SABD, schizoaffective bipolar disorder; and SCZ, schizophrenia.

Compared with controls, the PRSs were higher in BD, regardless of whether there was a history of psychosis (Table 1 and Figure 2). However, the PRSs were significantly higher in BD with psychosis, compared with BD without psychosis (Table 1 and Figure 3). Within BD cases, PRSs discriminated those with and without psychosis (RR = 1.25; 95% CI, 1.16-1.33; *P* < .001).

Post hoc analyses showed the association between PRS and psychosis was present in BD I (odds ratio [OR] = 1.21; 95% CI, 1.10-1.32) but was not statistically significant in BD II (OR = 0.98; 95% CI, 0.80-1.18). The composite subgroup, defined as BD I with psychosis, had higher PRSs compared with the PRSs in controls (RR = 1.38; 95% CI, 1.31-1.46). This association was significantly stronger than that of the composite BD I without psychosis (RR = 1.16; 95% CI, 1.08-1.25). Within BD II, there was no differential association across subgroups, defined by presence or absence of psychosis, as compared with the differential association in controls (eTable 2 in the Supplement).

### PRS Associations With Mood-Incongruent Psychotic Features

Psychotic BD characterized by high mood incongruence had a higher SCZ polygenic risk burden than that in controls, with a 1-SD increase in PRS increasing the RR of being in the high mood-incongruence category by 46% (RR = 1.46; 95% CI, 1.36-1.57) (Figure 3 and Table 1). Although the association was significantly weaker than for the high mood-incongruent group, SCZ risk alleles were enriched in those with low mood-incongruence, compared with controls (RR = 1.24; 95% CI, 1.17-1.33). Sensitivity analysis excluding the SABD group from analyses found comparable results (Table 1). Finally, a within-BD case analysis, measuring mood incongruence on an ordinal scale, found the odds of having higher levels of mood incongruence increased with increasing PRS (OR = 1.17; 95% CI, 1.08-1.27; *P* < .001). Analyses excluding the SABD sample found comparable results (OR = 1.20; 95% CI, 1.09-1.32; *P* < .001).

## Discussion

Higher PRS-SCZ in BD^20^ is well established. Here, we replicate and extend this observation, demonstrating a gradient of PRS associations across SCZ and BD subtypes (CLOZUK>SABD>BD I with psychosis>BD I without psychosis>BD II). In addition, we show that BD cases with psychosis carry a higher burden of SCZ risk alleles, compared with BD without a history of psychosis (RR = 1.09; 95% CI, 1.04-1.15). Furthermore, individuals with psychotic BD characterized by prominent mood-incongruent psychotic features carry the highest burden of schizophrenia risk alleles. There is a clear exposure-response gradient, with increasing PRS associated with psychotic BD and increasing mood incongruence (mood incongruent > mood congruent > no psychosis), supporting our hypothesis that mood-incongruence indexes phenotypic features linked to SCZ liability.

Previously published work examining the PRSs for SCZ across BD, stratified by psychosis, did not find significant discrimination,^22^ although a trend was observed that is consistent with the findings presented here. The most likely explanations for the enhanced signal in the current analysis are as follows: the PRSs were constructed using alleles derived from a larger SCZ-GWAS discovery set, which reduces the measurement error and improves power from both this sample and the larger BD sample.^61^ This group has shown that PRS-SCZ significantly differentiates SABD from non-SABD subtypes, while finding no statistically significant differential between BD stratified by psychosis,^40^ suggesting it is the nature of the psychotic symptoms rather than their presence that better indexes the liability shared with SCZ.^62^ The current analysis supports the proposition that it is the level of mood incongruence rather than the presence of psychosis that better specifies a shared biologically validated dimensional trait, which is captured, although with less precision, by the SABD diagnostic category.

Psychosis and mood-incongruent psychotic features are known to be correlated with poorer prognosis and treatment response.^30^ It is possible the transdiagnostic exposure-response gradient for the PRS, with the occurrence and nature of psychotic symptoms presented here, could be the result of a general psychopathological factor that cuts across psychiatric disorders and influences the severity of psychopathology generally as well as, or rather than, a psychosis-specific domain. The PRS derived from SCZ-GWAS may be indexing a general liability for psychopathological severity (at least in part)^63^ rather than a (SCZ) disease-specific liability.

### Implications

Our study supports the hypothesis that, within BD, positive and disorganized psychotic symptoms—particularly, mood-incongruent psychotic features—represent a dimensionally defined stratum with underpinning biological validity. These features are not only phenotypically similar to those observed in prototypal SCZ but also index a greater shared-genetic liability, which suggests BD and SCZ share more pathophysiological features.^64^ Notably, in those diagnosed with BD I with no history of psychosis, the association with SCZ liability was weaker but still higher than in the control group, while there was no overlap with SCZ liability in the BD II subsample. We are not suggesting that psychotic features are the best or the only index of shared pathophysiological features, but having established stronger genetic links between the risk for SCZ and BD characterized by the occurrence of psychosis and level of mood incongruence, we now have a basis to refine this signal. These findings represent a step toward the goal of reconceptualizing phenotypic definitions using richer clinical signatures, measured across quantitative or qualitative domains, including symptom loading and biomarker expression, outlined in the rationale for the RDC^65^ and the Road Map for Mental Health Research in Europe^67^ projects. However, a multidimensional stratification process will likely harness the observed clinical heterogeneity better and define more precise patient strata or subgroups in closer alignment with the underlying biological mechanisms.^68^

### Limitations

Phenotypic misclassification is a potential methodological concern. However, the phenotypic ratings used in the current analyses are based on both the SCAN interview and case-note review by raters with excellent interrater reliability, which is expected to minimize rates of missing data and differential misclassification due to recall bias of psychotic symptoms.^70^It is possible that differential misclassification of mood incongruence may still be present. The psychosis phenotypes examined in this study are broadly defined and likely to represent imperfect measurements of a phenotype that may be continuously distributed^71^; imposing categorical constraints as we have done may reduce power. Multiple testing can produce spurious results; thus, to reduce this likelihood we generated PRSs using a single discovery-set threshold of *P* < .05. Bootstrap resampling approaches were used within each of the 4 independent analyses to deal with multiple comparisons across different phenotypic strata. Bonferroni correction was used to adjust for possible familywise type I error proliferation. The PRSs were generated using the most probable genotypes that can potentially reduce power due to a (nondifferential) loss of information at some markers, making our results conservative. Cases and controls were collected independently, which can result in confounding due to population stratification and potential batch effects across the cases and controls. We mitigated against this by partialling out the first 10 principal components and genotyping platforms from the PRS, but some confounding is still possible. Finally, we have only examined the effect of common variants, as rare variants are not captured by current GWAS.

## Conclusions

To our knowledge, this study is the first to show a gradient of polygenic liability across SCZ and BD, indexed by the occurrence and level of mood incongruence of positive and disorganized psychotic symptoms. These results highlight the usefulness of genetic data to dissect clinical heterogeneity within and across disorders and suggest further research could potentially aid in defining patient stratifiers with improved biological precision and validity, moving us tentatively toward precision medicine in psychiatry.

## Notes

Supplement. **eAppendix.** Supplementary Notes **eTable 1.** Characteristics of Study Samples **eTable 2.** Differential Association of PRS Across BD Subtypes Stratified by Psychosis (Controls as Comparator Category)
