# Supplementary material for: Association Between Schizophrenia-Related Polygenic Liability and the Occurrence and Level of Mood-Incongruent Psychotic Symptoms in Bipolar Disorder
Source: JAMA Psychiatry. 2017 Nov 22;75(1):28–35. doi: 10.1001/jamapsychiatry.2017.3485 (PMC5833541; doi:10.1001/jamapsychiatry.2017.3485)
Supplement: Supplement. — eAppendix. Supplementary Notes eTable 1. Characteristics of Study Samples eTable 2. Differential Association of PRS Across BD Subtypes Stratified by Psychosis (Controls as Comparator Category) [file jamapsychiatry-75-28-s001.pdf]

Supplementary Online Content

Allardyce J, Leonenko G, Hamshere M, et al. Association between schizophrenia-related polygenic liability and the occurrence and level of mood-incongruent psychotic symptoms in bipolar disorder. *JAMA Psychiatry*. Published online November 22, 2017. doi:10.1001/jamapsychiatry.2017.3485

eAppendix. Supplementary Notes

eTable 1. Characteristics of Study Samples

eTable 2. Differential Association of PRS Across BD Subtypes Stratified by Psychosis (Controls as Comparator Category)

This supplementary material has been provided by the authors to give readers additional information about their work.

© 2017 Allardyce J et al. *JAMA Psychiatry*.

eAppendix. Supplementary Notes

eNote 1. Mood Congruence (Lifetime)

Using all available information from SCAN interview and case not review the rater makes a judgement on the overall balance between mood-congruent and mood-incongruent psychosis across the lifetime with very good inter-rater reliability kappa = 0.89.

0 No psychotic features

1 Virtually all content congruent with affective state

2 More congruent than not

3 Congruent and incongruent equally

4 More incongruent than congruent

5 Virtually all content incongruent

9 Unsure/unknown

© 2017 Allardyce J et al. *JAMA Psychiatry*.

eNote 2. Testing for Multiple Comparison Using Bootstrap Resampling with Replacement

Permutation methods, use existing data to create resampled data-sets, for example in logistic regression we shuffle the dichotomous outcome, to create many new simulated datasets. The justification for this reshuffling is that, this is what would be observed, if there was no association. This method guarantees a type 1 error control, under an exchangeability assumption [74] and this rationale extends to the bootstrap method used in this analyses, where we resample the observation vectors in memory intact, with replacement (so each observation could occur once, more than once or not at all – in a bootstrap replication sample of the same size as the original observed dataset) from the set of all observations instead of without replacement as in permutation based tests.

Using resampling with replacement allows us to maintain the strata and clusters within the variable structure of the MNLM, yet still approximating to permutation methods type 1 error control [75]. The variation across the replication samples, is used to estimate the standard deviation/variance of the sampling distribution. We drew 5000 bootstrap samples for all the MNLM and OLM presented[76].

Results

CLOZUK – Treatment resistant Schizophrenia, treated with clozapine, BD I - RDC bipolar disorder subtype I, BD II - RDC bipolar disorder type II, SABD-RDC bipolar schizoaffective disorder, LEP – lifetime ever occurrence of psychotic symptoms, LMI – lifetime pattern of low/high mood incongruent psychotic features RR – relative risk ratio PRS adjusted for 1st 10 PCs and genotyping platform

© 2017 Allardyce J et al. *JAMA Psychiatry*.

eTable 1. Characteristics of Study Samples

Diagnostic Category	Sample Size	% Total Sample
RDC BD I	2,775	15.09
RDC BD II	1,268	6.90
RDC SABD	356	1.94
CLOZUK	4,976	27.06
CONTROLS	9,012	49.01
TOTAL	18,387	100

© 2017 Allardyce J et al. *JAMA Psychiatry*.

eTable 2. Differential Association of PRS Across BD Subtypes Stratified by Psychosis (Controls as Comparator Category)

	N (subsample)	RR	p-value	95% Confidence Intervals
CLOZUK	4,976	1.94	<0.0001	1.86, 2.01
SABD	356	1.37	<0.0001	1.22, 1.54
BD I with psychosis	1807	1.38	<0.0001	1.31. 1.46
BD I without psychosis	949	1.16	<0.0001	1.08, 1.25
BD II with psychosis	138	1.01	0.87	0.84, 1.22
BD II without psychosis	1129	1.04	0.24	0.97, 1.11

© 2017 Allardyce J et al. *JAMA Psychiatry*.
